# Effects of Crude Polysaccharides from *Lentinula edodes* Obtained by Ultrasound-Assisted Water Extraction on Metabolic and Hepatic Parameters in Obese Rats

**DOI:** 10.3390/ijms27198631

**Published:** 2026-09-26

**Authors:** Nannapat Phosarith, Thanyaporn Siriwoharn, Wachira Jirarattanarangsri

**Affiliations:** Division of Food Science and Technology, Faculty of Agro-Industry, Chiang Mai University, Chiang Mai 50100, Thailand; nannapat_psr@hotmail.com

**Keywords:** shiitake, mushroom, crude polysaccharides, metabolic health, insulin sensitivity, dyslipidemia, rat, high-fat diet

## Abstract

A high-fat diet is associated with metabolic disorder, including obesity, insulin resistance, dyslipidemia, and hypertension. Polysaccharides derived from mushrooms have attracted interest for their potential metabolic and anti-inflammatory effects. Nevertheless, the impact of crude polysaccharides derived from the *Lentinula edodes* (Berk.) Pegler (PSC) on metabolic conditions linked to high-fat diets remains insufficiently studied. This study aimed to evaluate the bioactivity of PSC obtained using ultrasound-water extraction (UW) on metabolic parameters, hepatic function, and inflammation in rats fed a high-fat diet (HFD). Male Wistar rats (5 weeks old, 180–200 g; *n* = 6 per group) were assigned to normal-diet (ND) or high-fat-diet (HFD) groups, with or without PSC administration. PSC was administered by oral gavage at 400 mg/kg body weight/day for 4 weeks. Compared with the HFD group at week 20, the HFD+PSC group showed 30.44% lower body-weight gain, while LDL-C, AST, ALT, and IL-6 levels were 42.31%, 35.77%, 55.15%, and 41.78% lower. Histopathological examination showed reduced hepatic steatosis and inflammatory alterations following PSC extract administration, with the NAS decreasing from 5.60 ± 0.51 in the HFD group to 2.00 ± 0.32 in the PSC extract administration. The findings indicate that a PSC extract decreased various metabolic, hepatic, and inflammatory changes correlated with HFD. This study supports the potential of PSC extract as a mushrooms-derived functional ingredient to improve metabolic function.

## 1. Introduction

Metabolic syndrome (MS) is a group of metabolic disorders characterized by hyperglycemia, dyslipidemia, hypertension, and abdominal obesity. Each element of metabolic syndrome may elevate the risk of cardiovascular disease, diabetes, stroke, and mortality [1]. MS is an expensive healthcare condition that impairs patients’ quality of life, making it an important global problem in public health [2]. The increase in MS has been attributed to modern lifestyles that include lack of exercise, sedentary behavior, and high calorie diets, which are key factors related to the MS epidemic [3]. Strategies for decreasing MS include surgical intervention, pharmacotherapy, physical activity, and nutritional management. Weight loss surgery is the most rapid and effective treatment for MS [4,5]. However, the risk of postoperative complications, including acute hypoglycemia, dumping syndrome, and gastroesophageal reflux disease, limits the effectiveness of this therapy technique [6]. The long-term use of pharmacological therapies for metabolic disorders may be associated with limitations and potential negative effects. Therefore, lifestyle modifications, including regular physical activity and healthy dietary habits, are generally considered safer approaches for long-term management, although maintaining these behaviors may require substantial effort and sustained commitment. Consequently, functional foods have garnered increased interest in recent decades [7].

Polysaccharides extracted from the mushroom *Lentinula edodes* (Berk.) Pegler, known as shiitake mushrooms, have been investigated for their many bioactive properties [8]. Some research studies demonstrate anti-cancer properties [9]. *L. edodes* show antioxidant ability that enhance cellular antioxidant defense mechanisms [10]. Moreover, *L. edodes* has been recognized as a nutritious edible mushroom with potential health-enhancing properties involving antioxidant and anti-inflammatory effects, along with prospective advantages for lipid and metabolic wellness [11]. Polysaccharides may contribute to lipid metabolism through several mechanisms, including binding to bile salts, promoting the conversion of cholesterol to bile acids, and directly interacting with lipids and cholesterol [12]. Importantly, these biological properties are closely associated with the physicochemical and structural characteristics of polysaccharides, which can be substantially influenced by the extraction method [13]. Therefore, selecting a suitable extraction method is essential for obtaining polysaccharides with favorable properties and biological functions.

In our previous study, ultrasound-water extraction was optimized to obtain a β-glucan-rich crude polysaccharide preparation from *L. edodes* with favorable physicochemical properties and in vitro bioactivities [14]. Nonetheless, the impact of crude polysaccharides derived from *L. edodes*, especially those extracted using ultrasound-water methods, on metabolic abnormalities caused by a high-fat diet is still inadequately defined. This study advances previous extraction and in vitro research by evaluating the optimized PSC preparation in vivo in rats on a high-fat diet. We investigated its effect on weight gain, glucose and lipid metabolism, insulin resistance, hepatic function, and liver histopathology, including the NAFLD activity score. We hypothesized that the administration of PSC would reduce the metabolic and inflammatory disruptions as well as hepatic changes linked to a high-fat diet.

## 2. Results and Discussion

### 2.1. Component Analysis of Crude Polysaccharide from L. edodes

The UW analysis of the PSC extract indicated a total carbohydrate content of 64.70 ± 1.36 g/100 g and a total protein content of 2.23 ± 0.06 g/100 g, as illustrated in Table 1. The presence of protein at these concentrations is evidence of crude polysaccharide. We evaluated the characteristics of crude polysaccharides from the mushroom *L. edodes* based on previous studies and identified α- and β-glucans, protein, and minor quantities of phenolic compounds [14]. The overall properties and biological activity of the crude polysaccharides may be influenced by the presence of co-extracted proteins. However, the particular function of these proteins was not determined in this investigation.

The extract contained total glucan, β-glucan, and α-glucan in amounts of 37.33 ± 0.87, 34.51 ± 0.82, and 2.82 ± 0.08 g/100 g. Respectively, The process through which UW may release elevated concentrations of β-glucan has a direct connection to the acoustic cavitation effect, which comes from the collapse of microbubbles in the solvent, which generates micro-jets, shock waves, and high shear forces, thereby facilitating cell wall disruption and enhancing mass transfer rates [15].

### 2.2. Monosaccharide Composition of Crude Polysaccharide

HPLC analysis of the monosaccharide profile indicated that glucose was only one monosaccharide identified in the PSC extract, with a value of 39.91 ± 2.42 g/100 g. Identification was based on comparison of the retention time with that of the corresponding glucose standard, and the glucose peak was observed at approximately 6.9–7.0 min. A sharp early-eluting signal was observed at approximately 2–3 min in both the glucose standard (Figure 1A) and PSC sample (Figure 1B); therefore, this signal was not assigned as a monosaccharide. In the PSC sample, a broad unresolved signal was also observed between approximately 3 and 6 min, but its identity could not be confirmed as a specific monosaccharide under the analytical conditions used. The other investigated monosaccharides were not detected under the analytical conditions used. LOD and LOQ were not experimentally determined in the present HPLC analysis; therefore, no numerical LOD/LOQ values are reported. This finding suggests that the composition of the polysaccharide is primarily composed of glucose units, consistent with a glucan-dominant polysaccharide composition. The predominance of glucose in the monosaccharide composition (Figure 1B) was consistent with the glucan analysis of the PSC extract, which exhibited a higher level of β-glucan (34.51 g/100 g) compared to α-glucan (2.82 g/100 g) (Table 1). Therefore, β-glucan represented the primary glucan component of the extract.

The HPLC-determined glucose content was slightly higher than the total glucan content. This difference can be attributed to the different analytical principles and calculation procedures used. In the K-YBGL β-Glucan Assay Kit (Yeast and Mushrooms) (Megazyme, Bray, Ireland), Glucose is used as the calibration standard for the colorimetric assay, and the resultant value is converted into glucan following the manufacturer’s calculation protocol, which incorporates the 162/180 conversion factor for the transformation of free glucose to anhydroglucose. However, the glucose concentration measured via HPLC was calculated directly from an external calibration curve for glucose. Consequently, the HPLC glucose and the enzymatically measured glucan values are not directly comparable. The monosaccharide compositions of PSC extracts are consistent with previous studies that identified glucose as the exclusive monosaccharide in *L. edodes* polysaccharides [16]. However, other studies investigating ultrasound extraction have reported broader monosaccharide profiles, identifying glucose, galactose, mannose, and arabinose [17], as well as more complex mixtures that also include fucose, xylose, and rhamnose [18]. These variances may result from variations in the mushroom source and strain, the conditions of extraction, and the ultrasound energy utilized, which may affect the degradation of the mushroom cell wall and the release of polysaccharide-related components [19].

### 2.3. FTIR Analysis

The component analysis showed that the crude polysaccharide extract contained total carbohydrates and β-glucan, with glucose as the only detected monosaccharide. The FTIR spectrum of the UW-derived crude polysaccharide is demonstrated in Figure 2.

The broad and intense absorption band at 3417 cm^−1^ results from the O–H stretching vibration, suggestive of both intramolecular and intermolecular hydrogen bonding in polysaccharides. This aligns with previous studies that identified a wide band around 3383 cm^−1^ in polysaccharides extracted from *L. edodes*, indicating hydrogen bonding interactions across polymer chains. The faint band at 2917 cm^−1^ correlates to a nearby C–H stretching vibration at 2929 cm^−1^ and is recognized as a polysaccharide-specific absorption peak, indicating a distinct carbohydrate structure [20]. The absorption band at 1645 cm^−1^ may correspond to the C=O stretching vibration of the carboxylate group. Zhou et al. [21] indicated that the band at 1652 cm^−1^ results from C=O asymmetric and symmetric stretching vibrations. Conversely, the 1645 cm^−1^ position may indicate the existence of a minor quantity of protein through the amide I band, corroborating the protein analysis results obtained from the extract. The prominent absorption band at 1031 cm^−1^ is considered crucial for validating the polysaccharide structure, as it corresponds to the C–O–C stretching vibration of the glycosidic bond and the pyranose ring structure, perhaps signifying the pyranose form of the glucosyl unit. The band in this location confirms that the glucose units in this extract are in the glucopyranose configuration [22]. Although the FTIR spectrum was consistent with the characteristic functional groups of polysaccharides, FTIR alone is insufficient to exclude the presence of sulfated polysaccharides or other structurally distinct components.

### 2.4. Effect of Crude Polysaccharide on Body Weight

Experimental indicated that rats subjected to a high-fat diet (HFD) was slightly lower (15.95%) compared to control rats (ND) after a 16-week period of obesity induction, therefore validating the efficacy of the obesity induction process. Following a 4-week treatment of PSC extract administered at a dosage of 400 mg/kg body weight per day, the weight increase in the HFD+PSC-treated group was markedly less than that observed in the high-fat diet group (Figure 3).

The HFD+NSS group demonstrated the highest weight gain of 90.33 ± 17.69 g, while the HFD+PSC group exhibited a lower weight gain of 62.83 ± 3.68 g, indicating a 30.44% decrease in weight gain compared to the HFD+NSS group. This difference did not reach statistical significance (*p* = 0.056). During the 4-week treatment period, cumulative food intake was significantly lower in the HFD+PSC group than in the HFD+NSS group (267.33 ± 1.32 vs. 416.67 ± 2.10 g/cage; *p* < 0.05), corresponding to an approximately 35.8% reduction (Supplementary Table S1). The HFD+PSC group exhibited lower cumulative food intake than the HFD+NSS group during the treatment period. Therefore, the lower body weight gain observed following PSC treatment may be partly attributable to reduced food intake. Feed efficiency was also evaluated, with FER values of 35.85% and 33.78% in the HFD+NSS and HFD+PSC groups, respectively, with no significant difference between the groups (*p* > 0.05). Although the viscosity of polysaccharides may potentially contribute to satiety, the present study did not directly assess satiety or establish a viscosity-mediated mechanism. Comparable weight-related results have been reported for polysaccharides derived from *Pleurotus eryngii*. However, it is essential to account for variations in animal models, dosage, and treatment duration when comparing these results [23]. In the ND group, the ND+PSC group, and the ND+NSS group, comparable weight gain was observed, with no statistically significant differences among the groups (*p* > 0.05), suggesting that PSC administration did not significantly affect weight gain under standard dietary conditions.

### 2.5. Effect on Serum Lipid Profile

Serum triglyceride levels were evaluated to determine the effect of PSC extract on lipid accumulation caused by an HFD. The HFD+PSC group had a significant reduction in triglycerides of 57.00 ± 21.52 mg/dL according to Figure 4A, in contrast to the HFD+NSS group’s slight increase of 3.83 ± 16.74 mg/dL.

This finding is consistent with Lv et al. [24], who reported a similar reduction in triglyceride levels in HFD-fed rats after 8 weeks of treatment with *Ganoderma lucidum* polysaccharide extract (150 mg/kg body weight). Despite differences in mushroom species, quantities, and experimental durations, the results indicate a synergistic mechanism of action in the dietary fibers found in mushrooms, likely involving bile acid retention, diminished intestinal fat absorption, and inhibition of hepatic lipidosis [25]. The decrease in triglycerides is essential for preventing cardiovascular disease, since elevated triglyceride levels are an independent risk factor for hypertensive heart disease and metabolic syndrome [26]. The reduction in triglycerides was significantly correlated with the weight loss observed in the HFD+PSC group, which showed a 30.44% lower weight gain compared to the HFD+NSS group, indicating possible involvement related to the water holding and swelling properties of PSC extract, which may enhance satiety and subsequently reduce energy intake, thereby affecting fat accumulation. This hypothesis is supported by evidence from previous studies [14,27]. The ND+PSC group exhibited a triglyceride reduction of 18.67 ± 9.69 mg/dL, whereas the ND+NSS group demonstrated a reduction of only 3.00 ± 6.07 mg/dL. This data suggests that PSC extract effectively regulates triglyceride levels under normal conditions, with a more significant impact observed in groups with metabolic disorders.

The HFD+PSC group exhibited a notable reduction in Total cholesterol levels, amounting to 23.83 ± 8.21 mg/dL (27%), whereas the HFD+NSS group demonstrated an increase of 10.50 ± 6.32 mg/dL, as illustrated in Figure 4B. This illustrates the effectiveness of PSC extract in lowering cholesterol levels in rat subjected to an HFD. This outcome aligns with the discovery that PSC extract exhibits cholesterol-lowering attributes [12], associated with mechanisms of bile binding in the intestines and diminished fat absorption [28]. Our experimental results demonstrated greater total cholesterol-lowering effect compared to a previous study in which rats were given polysaccharides from the fruiting body of *Pleurotus eryngii* at a dosage of 200 mg/kg for 8 weeks, resulting in only a slight reduction in Total cholesterol levels [29]. This difference may result from many different factors including differences variations in polysaccharide composition, dosage and duration of treatment, and the origin of polysaccharide. The ND+PSC and ND+NSS groups exhibited comparable reductions in cholesterol. This finding indicated that, under standard conditions in the absence of an excessive lipid burden, β-glucan administration did not provide an additional cholesterol-lowering effect. This suggested that the PSC extract’s efficacy was more evident under hyperlipidemic conditions.

Low-density lipoprotein cholesterol (LDL), considered as an essential biomarker of atherosclerotic lipid profiles [30], was investigated. The HFD+PSC group showed a significant decrease in LDL of 9.33 ± 5.05 mg/dL, while the HFD+NSS group demonstrated an increase of 5.50 ± 2.59 mg/dL, as illustrated in Figure 4C. Reducing LDL is essential for lowering the risk of coronary artery disease and stroke, as LDL serves as a transporter of cholesterol that accumulates on arterial walls [31]. The decrease in LDL and triglycerides in the HFD+PSC group indicated a significant improvement in the overall lipid profile. This effect may be associated with the biological activities of the crude polysaccharide extract and its β-glucan-rich composition. However, the specific mechanisms underlying these effects were not directly investigated in the present study [14,32]. The ND+PSC and ND+NSS groups exhibited comparable reductions in LDL, suggesting that the impact of PSC extract is more significant in metabolic disorders.

High-density lipoprotein cholesterol (HDL) levels were evaluated to determine potential changes in lipid metabolism. The findings showed significant differences between the groups (*p* < 0.05). The HFD+PSC group exhibited an increase in HDL of 11.33 ± 2.27 mg/dL, whereas the HFD+NSS group demonstrated a decrease of 9.83 ± 3.56 mg/dL HFD+PSC, as illustrated in Figure 4D. Research indicates that polysaccharides derived from *Ganoderma lucidum* mushrooms may stimulate LXRα-ABCA1/ABCG1 signaling, therefore increasing HDL synthesis [33]. Increased HDL is an essential indicator of improved lipid metabolism. HDL supports reverse cholesterol transport by carrying excess cholesterol from peripheral tissues to the liver for elimination. This process contributes to a reduction in overall cholesterol levels [34]. The ND+NSS and ND+PSC exhibited comparable elevations in HDL (7.33 ± 3.71 and 6.33 ± 1.02 mg/dL), suggesting that HDL levels are markedly elevated under normal conditions. The administration of PSC did not have a significant effect on HDL levels (*p* > 0.05).

### 2.6. Effects on Glucose Homeostasis and Insulin Resistance

The experimental findings indicated that the HFD+PSC group exhibited a significant decrease in glucose levels of 29.44 ± 6.56 mg/dL, while the HFD+NSS group demonstrated an increase of 39.32 ± 12.9 mg/dL, as shown in Table 2. Because fasting glucose levels differed significantly between the HFD+NSS and HFD+PSC groups at baseline (week 16), ANCOVA was performed using the week-16 fasting glucose level as a covariate. Following the adjustment for the baseline value, the HFD+PSC group exhibited a significantly lower estimated marginal mean of fasting glucose at week 20 compared to the HFD+NSS group (96.29 ± 5.45 compared to 184.65 ± 7.50 mg/dL, accordingly; adjusted mean difference = 88.36 mg/dL, *p* < 0.001). In addition, fasting glucose level in the HFD+PSC group did not change significantly from that in the ND+NSS group (*p* = 0.589). These findings illustrate the effectiveness of PSC extract may contribute to the regulation of fasting glucose levels in HFD-fed rats after accounting for the baseline imbalance. The observed improvement in glucose-related measurements could be associated with the previously studied inhibitory effects of *L. edodes* crude polysaccharide on α-amylase (IC_50_ = 1.59 mg/mL) and α-glucosidase (IC_50_ = 2.49 mg/mL) [14]. The extract in Ref. [14] was obtained by using the optimized ultrasound-water extraction parameters employed in this study, offering preliminary proof for its possible glucose-regulating properties. Nonetheless, the activities of these enzymes were not directly evaluated in the present animal study and therefore persist as a potential mechanism. Inhibiting these enzymes reduces glucose absorption in the intestines, thereby reducing the postprandial glucose spikes and helping in the prevention of chronic hyperglycemia [35]. Moreover, raising the viscosity of food within the gastrointestinal tract slows the movement of glucose to the intestinal wall, decreasing the absorption rate [17]. This is the weight loss observed in the HFD+PSC group. In the standard diet groups, both ND+PSC and ND+NSS group exhibited a minor, non-significant decrease in glucose levels (*p* > 0.05), suggesting that β-glucan does not induce hypoglycemia under normal conditions.

The HFD+PSC group demonstrated a significant decrease (*p* < 0.05) in insulin levels of −32.29 ± 9.52 μU/mL, while the HFD+NSS group demonstrated an increase of 29.16 ± 9.57 μU/mL. The reduction in insulin levels, along with an associated decrease in glucose levels, serves as an essential indication of improved insulin sensitivity [36]. Elevated insulin levels in the HFD+NSS group, accompanied by high glucose levels, indicate insulin resistance resulting from prolonged consumption of an HFD. The body needs to produce additional insulin to regulate blood glucose levels [37]. In contrast, the HFD+PSC group demonstrated effective glucose regulation with reduced insulin levels, indicating improved cellular insulin sensitivity. The reduction in body-weight gain was accompanied by improved insulin sensitivity, as reflected by lower HOMA-IR values, together with an improved lipid profile, particularly reduced triglyceride levels and increased HDL-C levels. These effects may be related to mechanisms reported in previous studies [38], in which lipid accumulation in metabolic tissue has been linked to insulin resistance. Consequently, these reductions in adiposity facilitate the restoration of insulin functionality [33]. The normal diet group showed low insulin levels, suggesting that, in the lack of high adiposity, the glycemic control system functions effectively without demanding elevated insulin production.

Homeostatic Model Assessment for Insulin Resistance (HOMA-IR) is an index derived from fasting glucose and insulin concentrations to evaluate insulin resistance. An increased HOMA-IR result indicates increased insulin resistance [39]. Wistar rats fed a high-fat diet for 12–16 weeks generally show HOMA-IR values between 25 and 45 [40], in accordance with the values found in the HFD group of our study. The HFD+PSC group had a significant reduction in HOMA-IR at 16.72 ± 4.63, while the HFD+NSS group increased by 21.80 ± 6.54. The almost 46.16% reduction in HOMA-IR within the HFD+PSC group provides evidence of a physiologically meaningful improvement in insulin sensitivity, as illustrated in Table 3.

### 2.7. Effects on Liver Function Markers

The experimental results indicated that a significant reduction in AST levels was observed in the HFD+PSC group (37.27 ± 11.03 U/L). However, the HFD+NSS group exhibited an increase of 14.24 ± 5.94 U/L, as illustrated in Figure 5. The reduction in AST in the HFD+PSC group (37.27 ± 11.03 U/L) suggests improved liver function with levels approaching those observed in the ND group. AST is an enzyme present in the cells of the liver, heart, muscle, and kidneys. When these cells are compromised, AST permeates into the bloodstream, resulting in increased blood levels [41]. The increased AST values in the HFD+NSS group indicate liver cell damage due to hepatic steatosis, caused by prolonged high-fat dietary intake and insulin resistance [42].

The reduction in AST in the HFD+PSC group was accompanied by a decrease in triglycerides. Elevated triglycerides have been associated with hepatic lipid accumulation and liver dysfunction [43]. The combined changes found in the current investigation may suggest improvement in hepatic metabolic condition. However, a direct relationship between AST and triglyceride level was not evaluated. Typically, insulin resistance causes the liver to produce an excess of fat, especially derived from triglycerides. Enhancing insulin sensitivity reduces this process [44,45]. In addition, PSC extract helps control glucose levels, as persistently high blood sugar levels promote hepatic fat synthesis through lipid metabolism. Compared to study on administering 200–400 mg/kg of a polysaccharide isolated from *Ganoderma atrum* (PSG) to obese mice injected with streptozotocin (STZ) to induce diabetes, AST levels were significantly reduced, indicating that PSG partially alleviates the condition [46], reflecting the fact that maintaining normal blood sugar levels reduces the burden on the liver [47].The normal diet groups, ND+PSC and ND+NSS, exhibited slightly reduced AST levels within physiological levels, indicating normal liver function in the absence of metabolic stress.

The HFD+PSC group showed a significant decrease in ALT protein levels of 11.38 ± 19.31 pg/mL. In contrast, the HFD+NSS group showed an elevated level of 58.02 ± 22.87 pg/mL, as illustrated in Figure 6. This indicates a 55.15% increase compared to the HFD+PSC group. The elevation of ALT protein in the HFD+NSS group suggests impaired liver function associated with prolonged HFD consumption, along with insulin resistance and increased triglyceride levels. The reduction in ALT protein in the HFD+PSC group indicates decreased inflammation and hepatic injury, with PSC extract promoting the decrease in hepatic fat accumulation by improving lipid metabolism and insulin sensitivity. Additionally, the antioxidant characteristics of PSC extract protect hepatic cells from lipid peroxidation [48]. In rats on a normal diet, both the ND+PSC and ND+NSS groups exhibited no differences in ALT protein levels (*p* > 0.05), remaining within physiological levels. Although ALT protein is measured mainly by protein concentration rather than enzyme activity, increased serum ALT protein levels have been associated with hepatic cellular damage [47]. The concurrent reduction in AST activity and ALT protein levels indicates a potential preventive effect against liver damage induced by a high-fat diet.

### 2.8. Effects on Systemic Inflammation

The experimental results indicated that the HFD+PSC group had a significant reduction in IL-6 levels of 5.47 ± 2.08 pg/mL, which represents a decrease of approximately 19.7%. The HFD+NSS group had a notable increase of 9.83 ± 2.67 pg/mL (34.4% increase), as illustrated in Table 4. This significant difference demonstrates the effectiveness of PSC extract to control chronic inflammation induced by an HFD. IL-6 is a pro-inflammatory cytokine that is essential to both acute and chronic inflammatory processes associated with obesity and metabolic syndrome. Under conditions of excess adiposity, visceral adipose tissue releases elevated levels of IL-6, contributing to low-grade inflammation, which is a critical mechanism connecting obesity to type 2 diabetes, cardiovascular disease, and fatty liver disease [49].

The consistent increase in IL-6 in the HFD+NSS group demonstrates the development of metabolic inflammation. This initiates with continuous weight gain in the HFD+NSS group, resulting in the growth of adipose tissue, especially visceral fat, as adipocytes enlarge excessively. Hypertrophic adipocytes experience hypoxia and endoplasmic reticulum stress, resulting in elevated IL-6 release by invading adipocytes and macrophages [50]. Moreover, increased triglyceride levels and decreased HDL in the HFD+NSS group may induce atherogenic dyslipidemia, which triggers vascular inflammation and enhances IL-6 release from endothelial and vascular smooth muscle cells [51]. Increased AST levels, coupled with markedly higher ALT protein in the HFD+NSS group, signify hepatic damage and inflammation. Adipose and inflamed liver releases elevated levels of IL-6, facilitating the advancement from uncomplicated steatosis to non-alcoholic steatohepatitis (NASH) [52].

A significant decrease in IL-6 levels (19.7%) in the HFD+PSC group indicates the anti-inflammatory properties of the PSC extract [53]. Additionally, an enhanced lipid profile, specifically decreased triglyceride levels, led to a reduction in lipotoxicity and oxidative stress within adipose tissue, thus leading to lower inflammation [33]. The decrease in AST and ALT protein levels indicates a significant enhancement in liver health, as PSC extract decreases hepatic fat buildup by enhancing insulin sensitivity. It might decrease the activity of SREBP-1c, a crucial transcription factor in lipid biosynthesis [54]. The improvement in liver health immediately leads to a decrease in hepatic IL-6 secretion, a primary source of systemic IL-6. A previous study indicates that PSC extract comprises β-(1→3) glycosidic linkages, which specifically bind to Dectin-1 receptors on macrophages and dendritic cells. Binding to Dectin-1 may diminish the synthesis of pro-inflammatory cytokines, leading to reduced IL-6 levels [55]. The decrease in IL-6 levels found in this study may be attributed to the combined effects of multiple components. Beta-glucan (34% of the crude extract) is likely the principal contributor to the anti-inflammatory activity, as previous study [56] have reported its role in modulating immune responses through receptors such as Dectin-1. IL-6 was selected as a representative marker of obesity-related inflammation because of its established association with metabolic dysfunction. However, inflammation involves multiple cytokines and signaling pathways, and the assessment of IL-6 alone limits the interpretation of the anti-inflammatory effects of PSC. Future studies should evaluate additional inflammatory markers to provide a more comprehensive assessment.

### 2.9. Effects on Histopathology of Liver Tissue and NAFLD Activity Score

Histological evaluation utilizing H&E staining and NAFLD Activity Score (NAS) scoring demonstrated considerable improvement of liver morphology in the PSC extract group, as shown in Table 5. The steatosis score in the HFD+PSC group significantly decreased from 2.40 ± 0.54 to 1.00 ± 0.37 (*p* < 0.05), indicating a 58.3% reduction and an elevation from moderate to mild steatosis. The lobular inflammation score decreased by 50%, from 1.60 ± 0.55 to 0.60 ± 0.55. The hepatocellular ballooning score significantly lowered by 75%, from 1.60 ± 0.55 to 0.40 ± 0.55 (*p* < 0.05), indicating the most significant improvement among the three components of the NAS. The HFD+NSS group had a NAS of 5.60, indicating a definitive NASH classification (NAS ≥ 5), based on the total score of all three components. However, the HFD+PSC group exhibited a decreased NAS of 2.00 (NAS < 3), categorized primarily as non-NASH or simple steatosis.

The findings are consistent with histological analysis, which demonstrated that the HFD+NSS group exhibits severe macro vesicular steatosis, characterized by large lipid vacuoles displacing the nucleus laterally, along with scattered inflammatory foci and ballooned hepatocytes with pale, rarefied cytoplasm (Figure 7C). Conversely, the HFD+PSC group exhibited a marked decrease in lipid vacuoles, a more structured hepatic cord construction, and nearly normal hepatocyte morphology (Figure 7D).

Both ND groups exhibited total NAS values of 0.60–0.80 with normal hepatic structure, demonstrating that PSC extract does not induce liver disease. These histological improvements support and illustrate previously reported biochemical findings. The reduction in steatosis score is directly associated with the decrease in serum triglycerides and HOMA-IR, indicating that PSC extract may function through various components, including β-(1→3),(1→6)-glucan, the principal bioactive polysaccharide of shiitake mushrooms. This mechanism includes the formation of a viscous gel in the gastrointestinal tract that lowers fat absorption and enhances bile acid excretion in feces [57], as well as the suppression of hepatic de novo lipogenesis via improved insulin signaling [58]. The histology findings correlate with previous studies demonstrating a reduction in NAS in C57BL/6 mice following treatment with polysaccharide extract from Antrodia cinnamomea [59]. Moreover, polysaccharide extract from *Ganoderma lucidum* markedly decreased hepatic steatosis and lobular inflammation in C57BL/6N HFD mice [60]. Furthermore, the application of PSC extract in this study may suggest a synergistic interaction among the various constituents of the extracts. The mechanisms for reducing hepatocellular ballooning may involve diminished lipid accumulation, a significant catalyst for ER stress and mitochondrial impairment [61], alongside the antioxidant properties of PSC extract, validated by ABTS, DPPH, and FRAP assays as previously reported [14]. The decrease in lobular inflammation score was associated with a reduction in serum IL-6, aligning with findings that β-(1→3)-linked glucan structures may induce Dectin-1 receptors on Kupffer cells, facilitating M1-to-M2 macrophage polarization and suppressing NF-κB signaling [62,63]. Nonetheless, additional molecular investigations are required to validate these pathways.

Furthermore, in the ND+PSC group, the PSC extract had no significant effect on all parameters in comparison to the control group (ND+NSS) (*p* > 0.05). No significant differences were observed in weight increase, glucose and insulin levels, HOMA-IR, lipid profile, liver enzymes, or IL-6. This discovery supports the extract’s no induction of hypoglycemia, growth disruption, hepatotoxicity, or immunosuppression, as well as the specificity of its biological efficacy against metabolic diseases. The limitation of this current study is the limited structural analysis of PSC. Although the characterization of PSC is based on glucan content, monosaccharide composition, and their physical and chemical characteristics in our previous study [14], specific structural features, including molecular weight, branching degree, glycosidic linkage patterns, and molecular structure, remain to be further clarified. These structural attributes may influence the biological efficacy of polysaccharides and result in variations in physiological impacts. Therefore, the metabolic and inflammatory effects observed in the present study cannot be conclusively attributed to specific structural characteristics of PSC. Moreover, because PSC was obtained as a crude polysaccharide preparation, the contribution of other minor chemical components, including the possible presence of sulfated polysaccharides, cannot be completely excluded based on the current characterization. However, this study has several limitations. The study included only male rats, used a single dose of PSC, and had a relatively short treatment duration. Therefore, further studies involving both sexes, multiple doses, and longer treatment periods are warranted to further validate these findings.

## 3. Materials and Methods

### 3.1. Materials and Chemicals

Dried *L. edodes* mushrooms were obtained from a local market in Chiang Mai Province, Thailand. The dried mushrooms were crushed with a hammer mill and sieve to get particle sizes less than 0.5 mm. The dry powder was dissolved in distilled water at a ratio of 1:30 (*w*/*v*). The mixture performed ultrasonic treatment with a 25 mm probe (20 kHz frequency, 750 W peak power, model: SONIC 3, SONIC Corporation, Newtown, CT, USA) at 60% amplitude for 35 min. After treatment, samples were centrifuged at 8000 rpm for 10 min at 4 °C. The supernatant was mixed with 95% ethanol (1:2, *v*/*v*) and incubated at 4 °C overnight. After centrifugation under the same conditions, the crude extract precipitate was dried at 65 °C in a vacuum oven and stored at −18 °C. Following the procedure detailed in our previous research [14]. The samples were stored at −20 °C before testing in rats. For performing tests on rats, *L. edodes* polysaccharide samples were dissolved in water and heated at 60 °C for 30 min. The samples were homogenized with a high-speed grinder at 10,000 rpm (Ultra-Turrax^®^ T25, IKA^®^-Werke GmbH & Co. KG, Staufen, Germany) for 20 min. The samples were left to cool before feed in rats. The quantified β-glucan content of the crude extract was 34.51 g/100 g dry weight, corresponding to an equivalent dose of 138.04 mg β-glucan/kg body weight for the administered 400 mg crude extract/kg dose.

### 3.2. Total Carbohydrate Content, Total Protein Content and β-Glucan Content

The phenol–sulfuric acid and Bradford methods were utilized to determine the total carbohydrate and protein content of the polysaccharide [64,65]. D-glucose and bovine serum albumin (BSA) served as standards for each experiment.

β-glucans were determined using the K-YBGL β-glucan Assay Kit (Yeast and Mushrooms) (Megazyme, Bray, Ireland), following the manufacturer’s instructions.

### 3.3. Monosaccharide Composition

The monosaccharide composition was measured after acid hydrolysis. Following brief, a 0.10 g of the sample was hydrolysis with 5 mL of 2 M HCl through ultrasonic treatment at 80 °C for 30 min, subsequently heated in a boiling water bath for 1 h. The hydrolysate was neutralized to a pH of 6–7 using 2 M NaOH and thereafter diluted to a final volume of 25 mL with distilled water. The solution was combined with acetonitrile (1:1, *v*/*v*), centrifuged at 10,000 rpm for 10 min, and subsequently filtered over a nylon membrane before analysis. Agilent 1260 Infinity II HPLC system (Agilent Technologies, Santa Clara, CA, USA) analysis was conducted utilizing a Shodex Asahipak NH_2_P-50 4E column (4.6 × 250 mm, Showa Denko, Tokyo, Japan) at 30 °C, employing acetonitrile/water (70:30, *v*/*v*) as the mobile phase at a flow rate of 1.0 mL/min. The injection volume was 6 µL, and detection was performed via a refractive index detector. Monosaccharides were detected and measured utilizing external standards.

### 3.4. Fourier-Transform Infrared (FT-IR) Spectroscopy

FTIR spectra were acquired utilizing a JASCO FT/IR 4700 Spectrometer (JASCO Inc., Easton, MD, USA) operated in KBr mode. All data were collected in the region from 400 to 4000 cm^−1^.

### 3.5. Animals

Twenty-four 5-week-old male Wistar rats (180–200 g, at the start of the experiment) were used in this study. The rats were obtained from Nomura Siam International Co., Ltd. (Bangkok, Thailand). Sample size was calculated using G*Power (version 3.1.9.6; Heinrich-Heine-Universität Düsseldorf, Düsseldorf, Germany) based on data from Handayani et al. [66]. All animal procedures were approved by the Ethics Committee of the Laboratory Animal Center, Chiang Mai University, Thailand (approval no. 2568/RT0007, 30 May 2025). Healthy male Wistar rats within the initial weight parameters were included, while animals exhibiting acute illness, excessive weight loss, or failure to adapt during acclimatization were excluded. During the 20-week investigation, animals were observed daily for specific humane endpoints (exceeding a 20% body weight loss, extreme lethargy, or incapacity to reach food and water; none of the animals satisfied these conditions). Subsequent to blood sample, 2.0 mL of sterile normal saline was injected subcutaneously as a fluid replacement to avert dehydration and hypotension.

### 3.6. Experimental Design

The rats were kept in individual cages under conditions of 21 ± 1 °C, 50 ± 10% humidity, and a 12/12 dark/light cycle, with ab libitum access to food and water under constant environmental conditions for one week. Before the experiment, baseline body weights and blood samples were collected as described in Section 3.6 and animals were allocated based on initial body weight to ensure baseline comparability across experimental groups.

Weeks 1–16: The rats were divided into two groups.

Group 1 (ND, *n* = 12): a normal diet (Smart Heart Hamster Food, Complete and Balanced formula; 54.5% carbohydrates, 24.0% protein, 4.5% fat, 5.0% fiber, and 12.0% other components).

Group 2 (HFD, *n* = 12): a high-fat diet (HFD) formulated with lard based on Pratchayasakul et al. (2011), consisting of 57.60% kcal from fat, 26.45% protein, 14.27% carbohydrates, and 1.68% cholesterol [24].

The diets were provided for 16 weeks. Body weight and food consumption were recorded weekly. At the end of week 16, blood samples were collected following the sampling procedure outlined in Section 3.6. During weeks 17–20, rat receiving ND and HFD were divided into two groups: a control group receiving 0.9% normal saline solution (10 mL/kg (6 rats) and a group receiving 400 mg/kg of PSC (6 rats) for 4 weeks.

Group 1: ND + Normal Saline Solution, 0.9% concentration, Volume = 10 mL/kg

Group 2: ND + PSC, 40 mg/mL concentration, Dose = 400 mg/kg, Volume = 10 mL/kg

Group 3: HFD + Normal Saline Solution, 0.9% concentration, Volume = 10 mL/kg

Group 4: HFD + PSC, 40 mg/mL concentration, Dose = 400 mg/kg, Volume = 10 mL/kg

Body weight and food intake continued to be monitored weekly. NSS and PSC were administered once daily by oral gavage. At the end of week 20, all rats were euthanized for terminal sample collection [67].

### 3.7. Blood and Tissue Sample Collection

Collections of blood were collected at three periods: baseline (before dietary treatment), week 16, and week 20. Before each blood sample collection, the rats had a fasting period of 12 h. At baseline and week 16, blood samples were obtained from the tail tip, following the administration of a subcutaneous injection of 2 mL of 0.9% normal saline solution (NSS) for hydration purposes. At the end of week 20, the animals were euthanized with an intraperitoneal injection of thiopental sodium (200 mg/kg), and terminal blood samples were obtained from the abdominal vein. All collected blood samples were centrifuged at 2000 rpm at 4 °C for 10 min. The collected serum was preserved at −80 °C before the examination of lipid profiles, glucose, insulin, aspartate transaminase (AST), alanine transaminase (ALT), and interleukin-6 (IL-6). Furthermore, hepatic tissues were collected postmortem at week 20 and stored for later histological analysis. The researcher responsible for animal care, oral gavage, and sample collection was aware of the group allocation. Histopathological evaluation and NAS scoring were performed by an independent assessor who was blinded to the treatment groups. Biochemical analyses and ELISA measurements were performed with knowledge of the group allocation.

### 3.8. Serum Lipid Measurement

Serum lipid levels, including Total cholesterol, HDL, and triglycerides, were determined using a Dri-Chem NX 500 automated clinical chemistry analyzer (Fujifilm, Tokyo, Japan), according to the manufacturer’s guidelines. Analyses of LDL and glucose were measured directly, rather than calculated using the Friedewald equation, by the Center for Veterinary Clinical Pathology and Animal Health Innovation, Faculty of Veterinary Medicine, Chiang Mai University, using an automated chemistry analyzer, model BX-3010 (Sysmex, Kobe, Japan), in accordance with the laboratory’s standard procedures.

### 3.9. Serum Insulin Measurement

Serum insulin concentrations were measured using a Rat Insulin ELISA Kit (Millipore, Burlington, MA, USA). Serum samples and insulin standard were placed into microplates pre-coated with rat insulin-specific monoclonal antibodies. After incubation, biotin-attached detection antibodies were added. Based on horseradish peroxidase (HRP)-conjugated streptavidin, the enzymatic reaction was developed using the substrate 3,3′,5,5′-tetramethylbenzidine (TMB) and stopped with a stop solution. Absorbance was measured at 450 nm using a microplate reader. Insulin concentrations were calculated from the generated standard curves using a four-parameter logistic regression (4PL) model.

The HOMA-IR index evaluates insulin resistance using fasting blood glucose and insulin levels by this formula in Equation (1).

(1) $$\text{HOMA-IR}=\frac{\mathrm{Fasting}\;\mathrm{Glucose}\;(\mathrm{mg}/\mathrm{dL})\times \mathrm{Insulin}\;\mathrm{level}\;(\mu U/\mathrm{mL})}{405}$$

The HOMA-IR value in rat models serves only as a relative comparison between experimental groups; it is not suitable for direct comparison with clinical criteria defined for humans.

### 3.10. Serum AST Activity Assay

Serum AST activity was determined using a Rat AST Assay Kit (Sigma-Aldrich, St. Louis, MO, USA) via enzymatic kinetic methods. Changes in absorbance were kinetically examined using a microplate reader, and AST activity was calculated according to the manufacturer’s instructions and expressed as U/L.

### 3.11. Serum ALT Protein Measurement

Serum ALT protein levels were determined using a Rat ALT ELISA Kit (Abcam, Cambridge, UK), which measures ALT protein concentration. Following incubation with HRP-conjugated antibodies and TMB substrate, absorbance was measured at 450 nm. ALT concentrations were determined from a standard curve with 4PL regression analysis.

### 3.12. Serum IL-6 Measurement

Serum IL-6 levels were measured using a Rat IL-6 ELISA Kit (Sigma-Aldrich, St. Louis, MO, USA) according to the manufacturer’s protocol. The assay used a sandwich ELISA design, and color development was performed using a TMB substrate. Absorbance values were read at 450 nm, and IL-6 concentration was calculated using 4PL curve modulation.

### 3.13. Histological Examination

For hematoxylin and eosin (H&E) staining, the formalin-fixed tissues were processed according to standard protocol. a standard paraffin-embedding protocol and sectioned at a thickness of approximately 4–5 µm. Histological assessment was performed using the NAFLD activity score (NAS) according to standard protocol [68], which includes steatosis (0–3), lobular inflammation (0–3), and hepatocellular ballooning (0–2), with a total score ranging from 0 to 8. Evaluation was conducted independently by five observers blinded to the experimental groups. The final NAS for each sample was calculated as the mean of the five independent assessments. Prior to analysis, all observers were calibrated to ensure consistency in scoring criteria.

### 3.14. Statistical Analysis

Data are presented as means ± standard error of the mean (SEM). The experiment used a randomized complete block design (RCBD). Baseline fasting glucose levels at week 16 were used as a covariate in an analysis of covariance (ANCOVA) to account for baseline differences, and estimated marginal means were compared using pairwise comparisons at a significance level of *p* < 0.05. Other parameters were analyzed using one-way analysis of variance (ANOVA) followed by Tukey’s honestly significant difference (HSD) test at a significance level of *p* < 0.05. Statistical analysis was performed using SPSS version 17.0 (SPSS Inc., Chicago, IL, USA).

## 4. Conclusions

This research demonstrates that crude polysaccharides derived from *Lentinula edodes*, obtained using an optimized ultrasound with a water extraction method, may enhance metabolic and liver cell results and decrease systemic inflammation in rats on a high-fat diet. These findings suggest initial in vivo proof supporting the potential of this crude polysaccharide as a factor in metabolic health. However, limitations on the study of single doses, inflammatory profiling, structural investigations, and reliance on animal models limit the relevance of these results to animal studies. Additional research is required to clarify the structure-activity relationship and the fundamental mechanisms of the active polysaccharide components and to assess their efficacy and safety in humans.

## Figures and Tables

**Figure 1:**
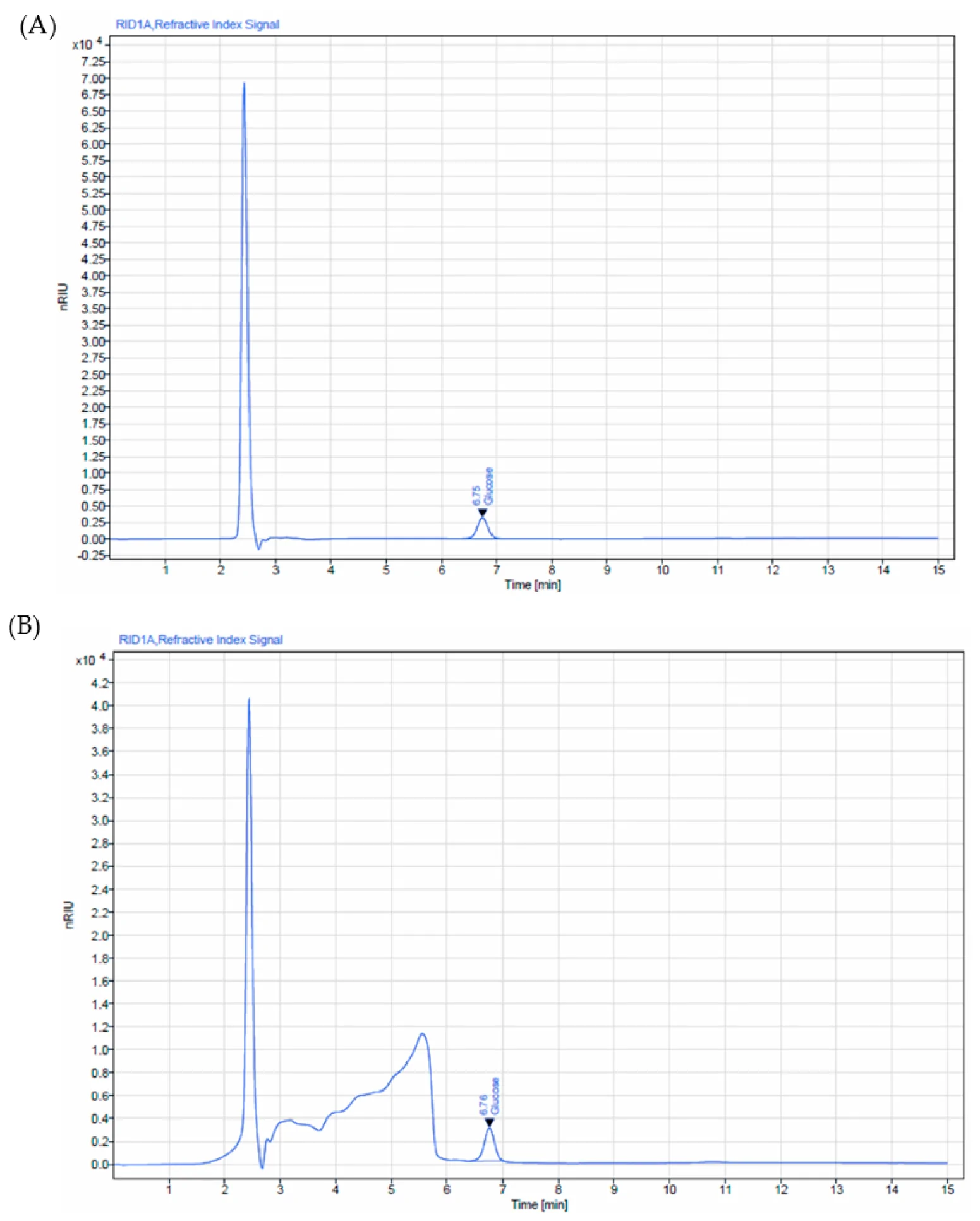
HPLC chromatograms of (**A**) glucose standard and (**B**) crude polysaccharide extracted from *L. edodes* using ultrasound-assisted water extraction (UW).

**Figure 2:**
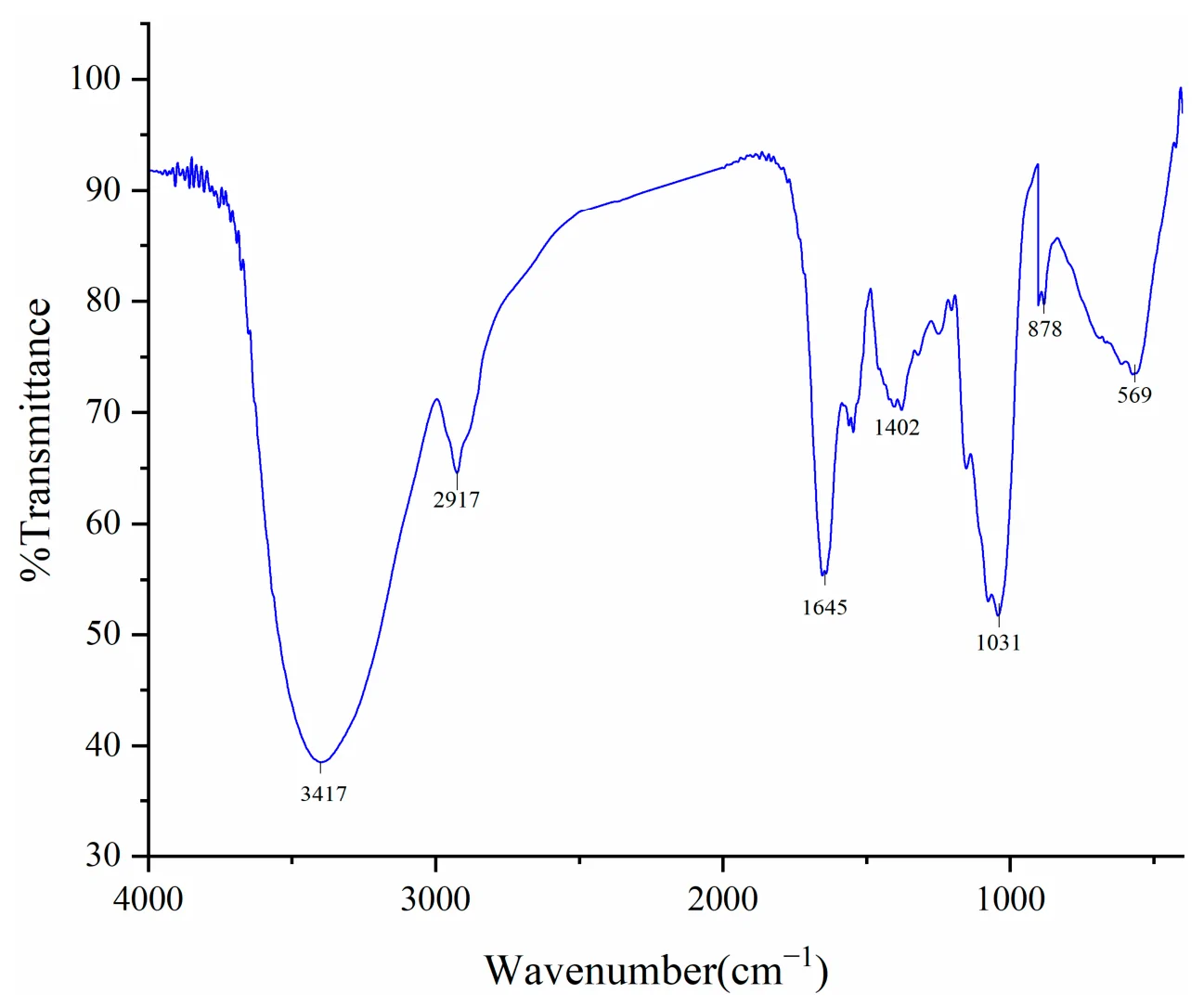
FTIR spectrum of isolated crude polysaccharides.

**Figure 3:**
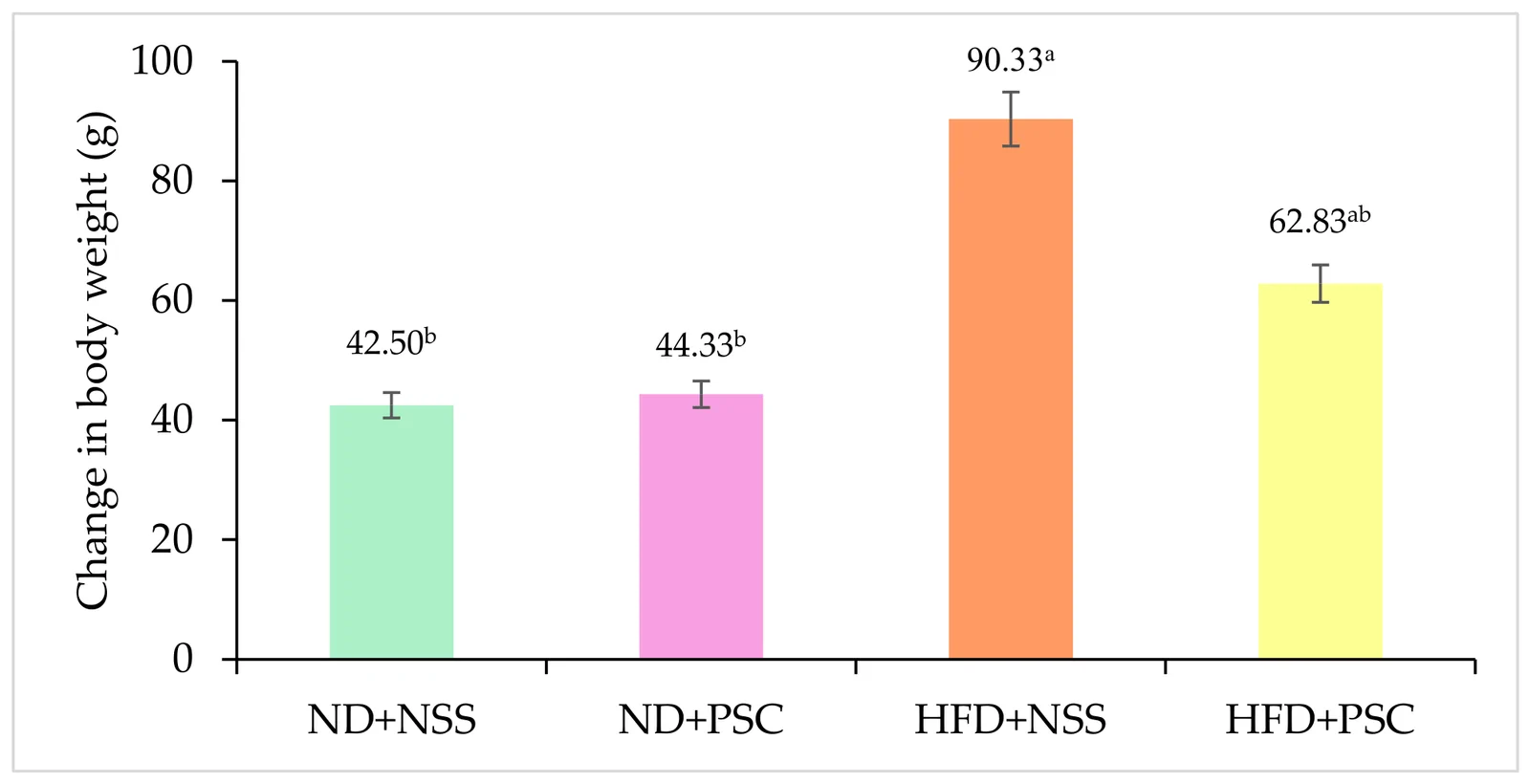
Body-weight gain of rats during the 4-week treatment period. Body-weight gain was calculated as the difference between body weight at week 20 (end of the treatment period) and body weight at week 16 (immediately before treatment), i.e., week 20 − week 16. HFD = high-fat diet; ND = normal diet; NSS = normal saline solution; PSC = crude polysaccharide extract from *L. edodes*. Different letters (a, b) above the bars indicate significant differences (*p* < 0.05) according to Tukey’s HSD test.

**Figure 4:**
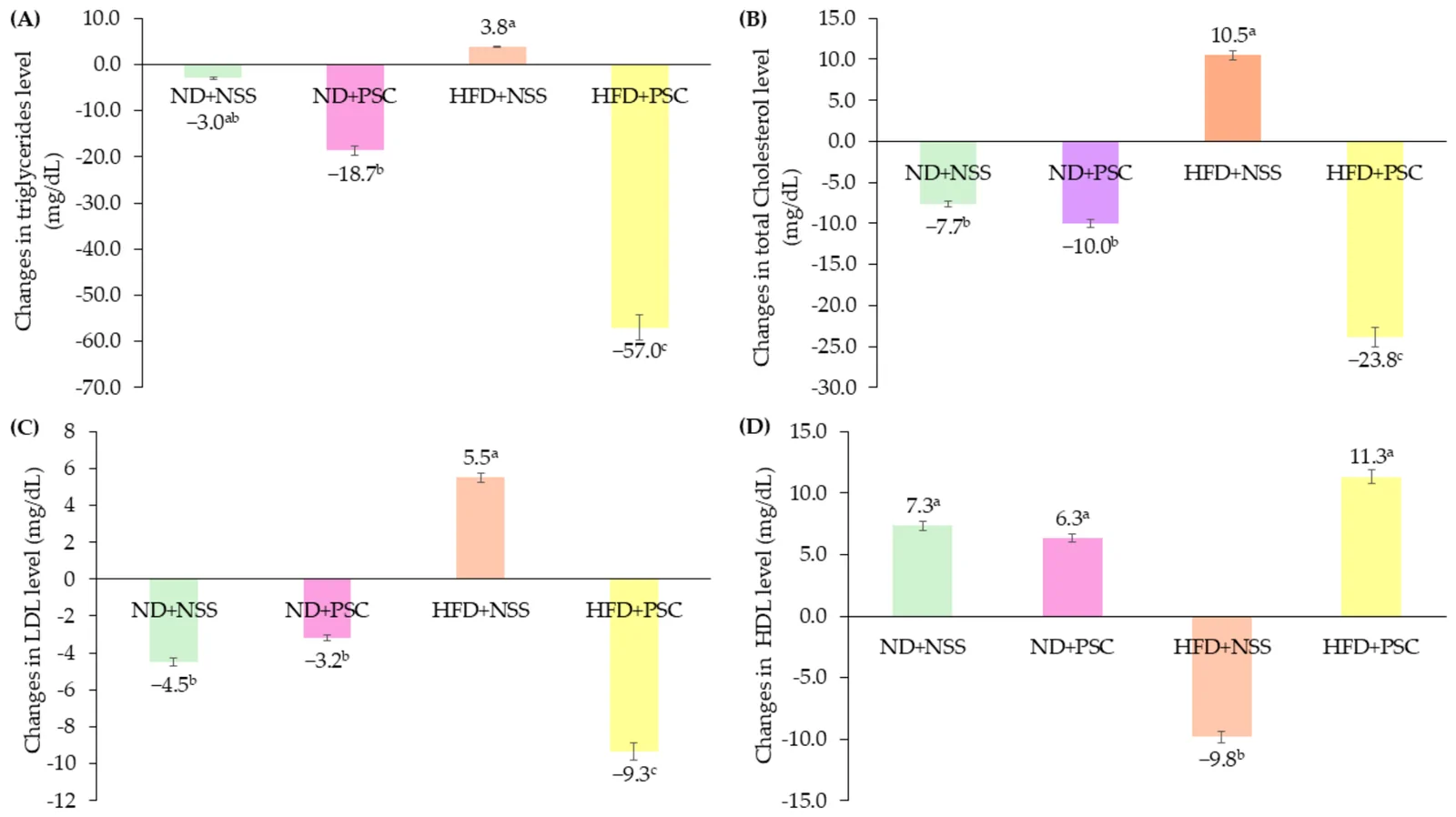
Changes in lipid profile parameters from week 16 to week 20 in the ND+NSS, ND+PSC, HFD+NSS, and HFD+PSC groups: (**A**) triglycerides (TG), (**B**) total cholesterol (TC), (**C**) LDL-C, and (**D**) HDL-C. Changes were calculated as week 20 minus week 16. Data are presented as mean ± SEM (*n* = 6 per group). HFD = high-fat diet; ND = normal diet; NSS = normal saline solution; PSC = crude polysaccharide extract from *L. edodes*. Different letters (a–c) above the bars indicate significant differences (*p* < 0.05) according to Tukey’s HSD test.

**Figure 5:**
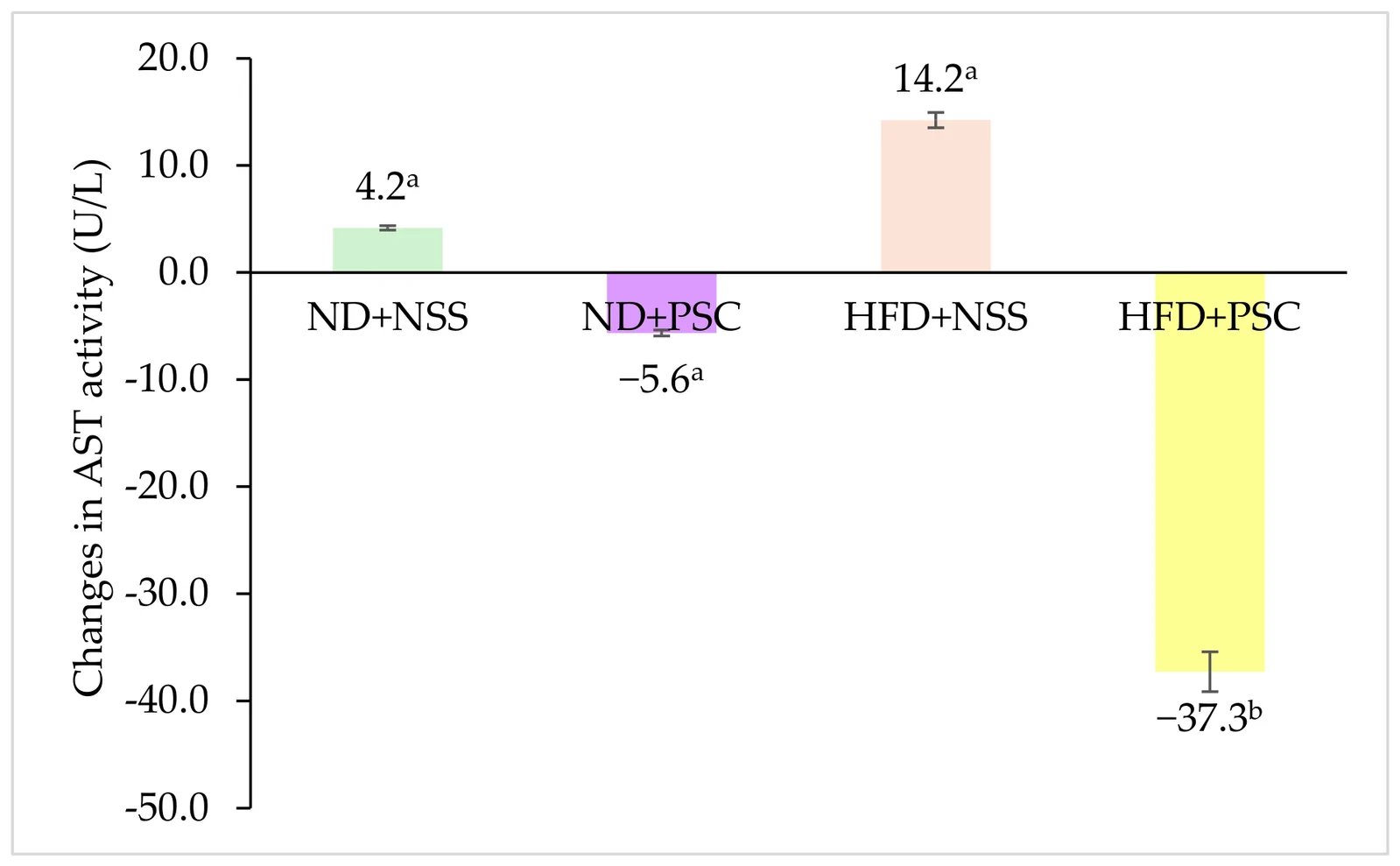
Changes in AST activity of rats from week 16 to week 20 in the ND+NSS, ND+PSC, HFD+NSS, and HFD+PSC groups. Changes were calculated as week 20 minus week 16. Data are presented as mean ± SEM (*n* = 6 per group). HFD = high-fat diet; ND = normal diet; NSS = normal saline solution; PSC = crude polysaccharide extract from *L. edodes.* Different letters (a, b) above the bars indicate significant differences (*p* < 0.05) according to Tukey’s HSD test.

**Figure 6:**
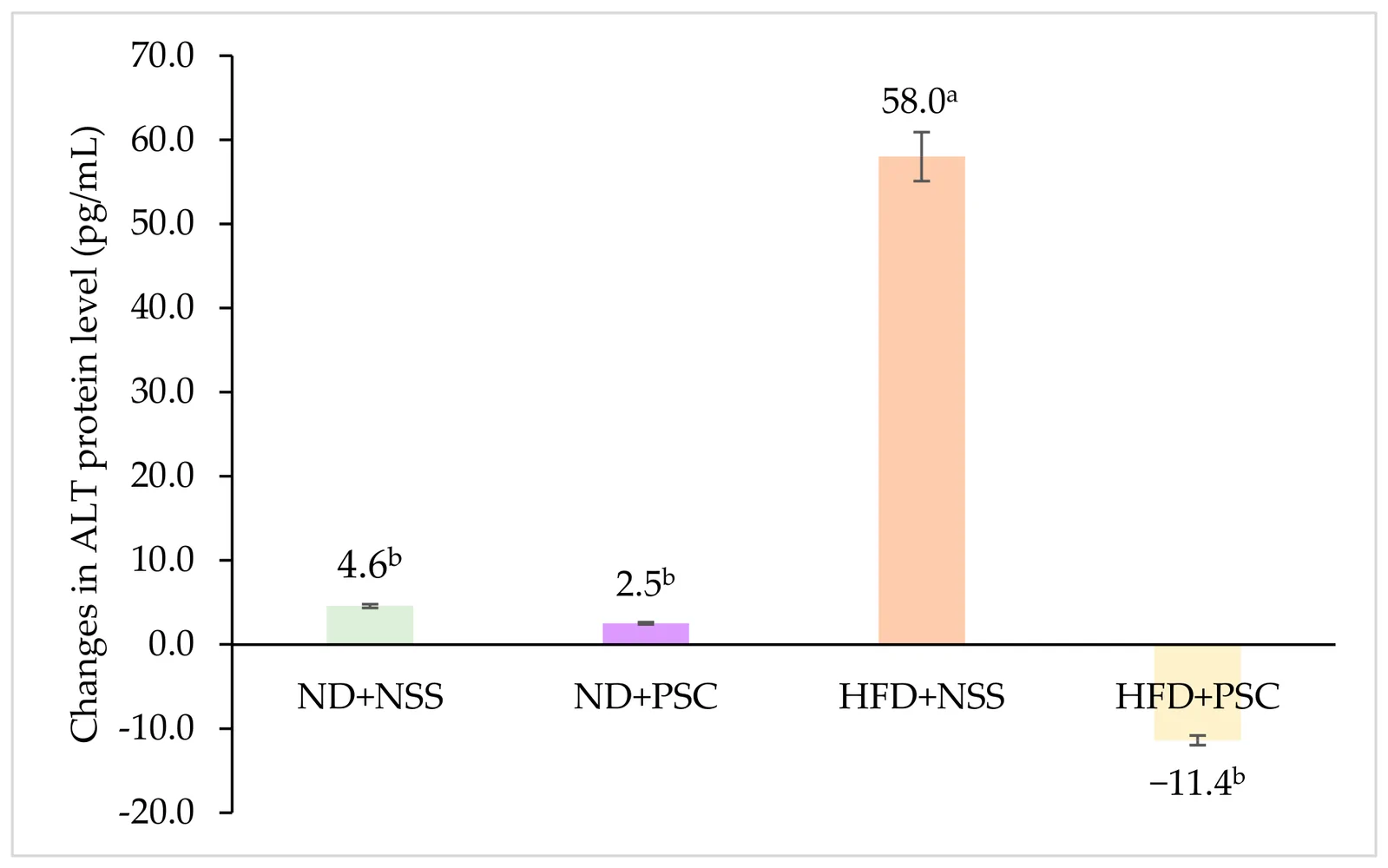
Changes in ALT protein of rats from week 16 to week 20 in the ND+NSS, ND+PSC, HFD+NSS, and HFD+PSC groups. Changes were calculated as week 20 minus week 16. Data are presented as mean ± SEM (*n* = 6 per group). HFD = high-fat diet; ND = normal diet; NSS = normal saline solution; PSC = crude polysaccharide extract from *L. edodes*. Different letters (a, b) above the bars indicate significant differences (*p* < 0.05) according to Tukey’s HSD test.

**Figure 7:**
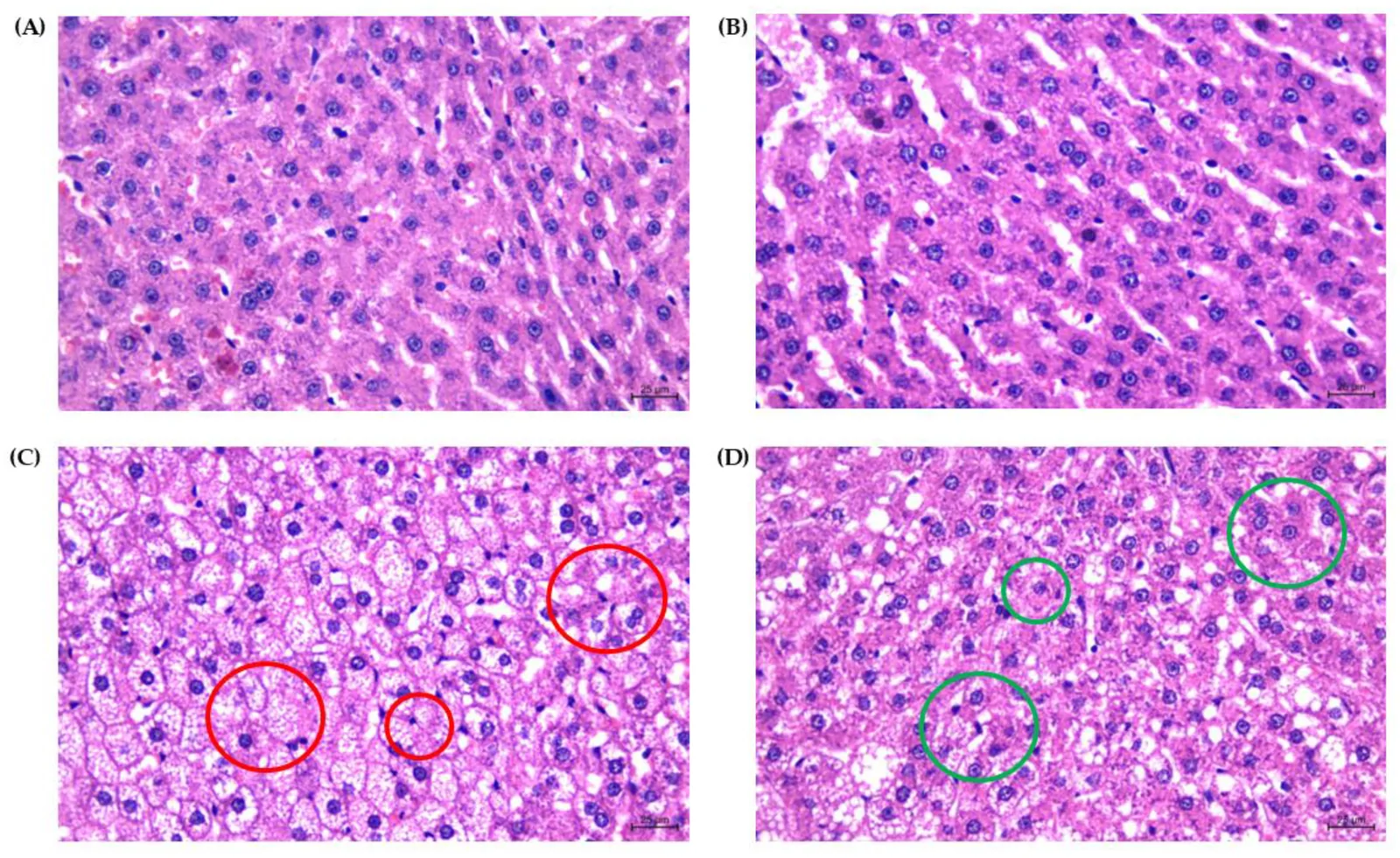
Examples of representative histopathological sections from livers sampled from rats in different experimental groups. (**A**) ND+NSS, (**B**) ND+PSC, (**C**) HFD+NSS, and (**D**) HFD+PSC. HFD = high-fat diet; ND = normal diet; NSS = normal saline solution; PSC = crude polysaccharide extract from *L. edodes*. Red circle indicate representative large lipid vacuoles associated with macrovesicular steatosis in the HFD+NSS group, whereas green circle indicate representative lipid vacuoles remaining in the HFD+PSC group. The HFD+PSC group exhibited fewer lipid vacuoles and improved hepatic morphology compared with the HFD+NSS group. Scale bar = 25 μm.

**Table 1:** Chemical composition of crude polysaccharide extracted from *L. edodes* by ultrasound-assisted water extraction (UW), including total carbohydrate content, total protein content, and β-glucan fractions (total glucan and α-glucan).

Polysaccharide Extracts	Total Carbohydrate Content (g/100 g)	Total Protein Content (g/100 g)	Glucan (g/100 g)		
			Total	α	β
*Lentinula edodes*	64.70 ± 1.36	2.23 ± 0.06	37.33 ± 0.87	2.82 ± 0.08	34.51 ± 0.82

Values are expressed as mean ± standard deviation (SD) of triplicate determinations (*n* = 3).

**Table 2:** Effects of PSC on fasting glucose and insulin levels in high-fat diet–fed and normal diet rats.

Experimental Group	Glucose Level (mg/dL)			Insulin Level (μU/mL)		
	Before Treatment (Week 16)	After Treatment (Week 20)	Change	Before Treatment (Week 16)	After Treatment (Week 20)	Change
ND+NSS	84.56 ± 2.78 ^c^	90.87 ± 6.64 ^a^	9.01 ± 3.58 ^b^	26.68 ± 7.43 ^b^	30.73 ± 5.58 ^c^	4.05 ± 9.63 ^b^
ND+PSC	89.19 ± 3.75 ^c^	75.35 ± 6.10 ^b^	−11.66 ± 6.21 ^bc^	30.32 ± 4.69 ^b^	28.10 ± 4.55 ^c^	−2.22 ± 0.79 ^b^
HFD+NSS	141.94 ± 9.74 ^a^	184.65 ± 7.50 ^c^	39.32 ± 12.99 ^a^	103.46 ± 12.62 ^a^	132.61 ± 7.21 ^a^	29.16 ± 9.57 ^a^
HFD+PSC	124.22 ± 5.17 ^b^	96.29 ± 5.45 ^a^	−29.44 ± 6.56 ^c^	115.95 ± 11.68 ^a^	83.66 ± 5.07 ^b^	−32.29 ± 9.52 ^c^

Data are expressed as mean ± SEM (*n* = 6). For week 20 fasting blood glucose, values represent estimated marginal means ± SE adjusted for week 16 baseline values using analysis of covariance (ANCOVA). Baseline data, week 20 serum insulin levels, and other biochemical parameters were analyzed using one-way ANOVA followed by Tukey’s HSD test. For the ANCOVA-adjusted week 20 fasting blood glucose, pairwise comparisons were performed at a significance level of *p* < 0.05. Different superscript letters (a–c) within the same column indicate statistically significant differences (*p* < 0.05). HFD = high-fat diet; ND = normal diet; NSS = normal saline solution; PSC = crude polysaccharide extract from *L. edodes*.

**Table 3:** Effects of PSC extract on insulin resistance (HOMA-IR) in high-fat diet–fed and normal diet rats.

Experimental Group	Before Treatment (Week 16)	After Treatment (Week 20)	Change
ND+NSS	5.53 ± 1.59 ^b^	7.16 ± 1.45 ^c^	1.63 ± 2.26 ^b^
ND+PSC	6.66 ± 1.03 ^b^	5.10 ± 0.58 ^c^	−1.56 ± 0.57 ^b^
HFD+NSS	37.40 ± 6.49 ^a^	59.20 ± 2.95 ^a^	21.80 ± 6.54 ^a^
HFD+PSC	36.24 ± 5.53 ^a^	19.51 ± 1.32 ^b^	−16.72 ± 4.63 ^c^

Data are expressed as mean ± SEM (*n* = 6 rats per group). HFD = high-fat diet; ND = normal diet; NSS = normal saline solution; PSC = crude polysaccharide extract from *L. edodes*. Different letters (a–c) within the same column indicate significant differences (*p* < 0.05) according to Tukey’s HSD test.

**Table 4:** Effects of PSC extract on IL-6 level in high-fat diet–fed and normal diet rats.

Experimental Group	IL-6 Levels (pg/mL)		
	Before Treatment (Week 16)	After Treatment (Week 20)	Change
ND+NSS	5.75 ± 3.49 ^b^	6.71 ± 2.42 ^c^	0.96 ± 5.01 ^ab^
ND+PSC	5.29 ± 2.84 ^b^	4.41 ± 2.83 ^c^	−0.88 ± 2.59 ^b^
HFD+NSS	28.56 ± 7.67 ^a^	38.39 ± 6.32 ^a^	9.83 ± 2.67 ^a^
HFD+PSC	27.83 ± 3.19 ^a^	22.35 ± 3.34 ^b^	−5.47 ± 2.08 ^b^

Data are expressed as mean ± SEM (*n* = 6 rats per group). HFD = high-fat diet; ND = normal diet; NSS = normal saline solution; PSC = crude polysaccharide extract from *L. edodes*. Different letters (a–c) within the same column indicate significant differences (*p* < 0.05) according to Tukey’s HSD test.

**Table 5:** Effects of PSC extract on NAFLD activity score (NAS) in high-fat diet–fed and normal diet rats.

Group	Steatosis	Lobular Inflammation	Hepatocellular Ballooning	NAS Total
ND+NSS	0.20 ± 0.45 ^c^	0.20 ± 0.45 ^c^	0.20 ± 0.45 ^b^	0.60 ± 0.24 ^c^
ND+BG	0.40 ± 0.55 ^c^	0.20 ± 0.45 ^c^	0.20 ± 0.45 ^b^	0.80 ± 0.37 ^c^
HFD+NSS	2.40 ± 0.54 ^a^	1.60 ± 0.55 ^a^	1.60 ± 0.55 ^a^	5.60 ± 0.51 ^a^
HFD+BG	1.00 ± 0.37 ^b^	0.60 ± 0.55 ^ab^	0.40 ± 0.55 ^b^	2.00 ± 0.32 ^b^

Data are expressed as mean ± SEM (*n* = 6 rats per group). NAS includes steatosis (0–3), lobular inflammation (0–3), and hepatocellular ballooning (0–2). HFD = high-fat diet; ND = normal diet; NSS = normal saline solution; PSC = crude polysaccharide extract from *L. edodes*. Different letters (a–c) within the same column indicate significant differences (*p* < 0.05) according to Tukey’s HSD test.

## Data Availability

The original contributions presented in this study are included in the article/Supplementary Materials. Further inquiries can be directed to the corresponding authors.

## Supplementary Materials

The following supporting information can be downloaded at: https://www.mdpi.com/article/10.3390/ijms27198631/s1.

## Abbreviations

The following abbreviations are used in this manuscript:

PSC	Crude polysaccharides derived from *Lentinula edodes* (Berk.). Pegler
HFD	High-fat diet
NSS	Normal saline solution
ND	Normal diet
