# Supplementary material for: Effects of Crude Polysaccharides from Lentinula edodes Obtained by Ultrasound-Assisted Water Extraction on Metabolic and Hepatic Parameters in Obese Rats
Source: Int J Mol Sci. 2026 Sep 26;27(19):8631. doi: 10.3390/ijms27198631 (PMC13665417; doi:10.3390/ijms27198631)
Supplement: Supplementary file 1 [file ijms-27-08631-s001.zip › ijms-4562766-supplementary.pdf]

Table S1. Cumulative food intake and feed efficiency ratio per rat during the 4-week treatment period (Weeks 17–20).

Experimental group	Food intake (grams/rat)	Feed efficiency (%)
	During treatment (week 17-20)	During treatment (week 17-20)
ND+NSS	871.83 ± 23.54[a]	4.72 ± 0.72[b]
ND+PSC	879.50 ± 39.81[a]	3.95 ± 0.46[b]
HFD+NSS	208.35 ± 2.10[b]	35.85 ± 8.51[a]
HFD+PSC	133.67 ± 1.32[c]	33.78 ± 1.42[a]

Food intake values are presented as cumulative intake per cage (2 rats per cage). Feed efficiency ratio (FER, %) was calculated per animal based on normalized individual food intake: FER (%) = [Body weight gain per rat (g) / Cumulative food intake per cage (g) / 2)] × 100.
